# Self-Assembly of a Purely Organic Bowl in Water via Acylhydrazone Formation

**DOI:** 10.3390/molecules28030976

**Published:** 2023-01-18

**Authors:** Guangcheng Wu, Tianyu Jiao, Hao Li

**Affiliations:** Department of Chemistry, Zhejiang University, Hangzhou 310027, China

**Keywords:** self-assembly, dynamic covalent chemistry

## Abstract

A bowl-shaped molecule can be self-assembled by condensing a triscationic hexaaldehyde compound and three equiv. of a dihydrazide linkers in pure water. The molecular bowl is thus composed of a triscationic π-electron deficient platform, as well as a hexagonal rim that contains six acylhydrazone functions. When the counteranions are chloride, the solid-state structure reveals that this molecular bowl undergoes dimerization via N–H···Cl hydrogen bonds, forming a cage-like dimer with a huge inner cavity. This molecular bowl can employ its cavity to accommodate a hydrophobic guest, namely 1-adamantanecarboxylic acid in aqueous media.

## 1. Introduction

Synthesizing cyclic host molecules and using their pockets or cavities for guest recognition have attracted great attention in the community of host-guest chemistry [1,2]. These hosts are often in the form of rings [3,4], cages [5], and bowls. In the literature, even although a number of cyclic molecules including cyclodextrins(CDs), [6,7,8] calixrenes(CAs) [9], and resorcin[n]arenes [10,11,12,13,14,15], as well as a variety of metallocavitands [16,17,18,19,20,21,22,23,24,25], have been claimed as bowl-shaped hosts, in fact, they are topologically not different from rings, except that the “peripheral walls” of these bowls have two rims with different sizes. A veritable molecule bowl is supposed to contain a bottom platform, on which a macrocyclic peripheral wall is grafted [26]. This host is therefore able to take advantage of both the “platform” and the “peripheral wall” to provide noncovalent interactions to bind guests. Compounds that best fit the definition of a bowl-shape should be curved π-conjugated molecules such as buckybowls, namely corannulene, sumanene, etc. [27,28,29], in which the bottom and the edges of the bowl structure are seamlessly merged. The derivatives of such bowl-shaped molecules have shown moderate affinity towards fullerenes which takes advantage of shape complementarity as well as concave-convex π-π interaction [30,31,32]. The preparation and functionalization of this kind of bowl-shaped conjugated molecules, however, are time-consuming and suffered from low yields. In order to obtain host molecules containing purely organic elements (i.e., C, H, O and N) with decent yields without the need of tedious synthetic procedures, chemists also developed dynamic covalent approaches [33,34,35,36,37,38,39,40] relying on reversible organic reactions. For example, disulfide bond formation was employed by Otto et al. [41,42,43,44,45,46] to accomplish self-assembly in weakly basic aqueous media. Imine [47,48,49,50,51,52,53,54] formation, has been considered as one of more often used dynamic approaches, because its precursors, namely aldehydes and amines are relatively more synthetic accessible, compared to thiol derivatives in disulfide approaches. Unfortunately, this labile bond is apt to undergo hydrolysis in water and therefore not amenable to use in aqueous media. This intrinsic drawback could be overcome by using an α-substituted imine, namely acylhydrazone [55,56,57,58,59]. This more robust dynamic bond has been used in the self-assembly of rings [60,61,62,63,64,65], cages [66,67,68,69,70], catenanes [37,71,72] as well as knots [73]. We thus envision that it might be possible to obtain bowl via acylhydrazone condensation.

Herein, by condensing a triscationic hexaformyl precursor and a bishydrazide in water, a purely organic triscationic bowl was self-assembled in a [1 + 3] manner. In solid state, two bowl molecules form a dimer, driven by the hydrogen bonding interactions between the amide functions of the rim of each bowl and the chloride couteranions. The bowl is capable of accommodating a sparingly soluble guest, namely 1-adamantanecarboxylic acid in water. 

## 2. Results and Discussion

**Figure 1:**
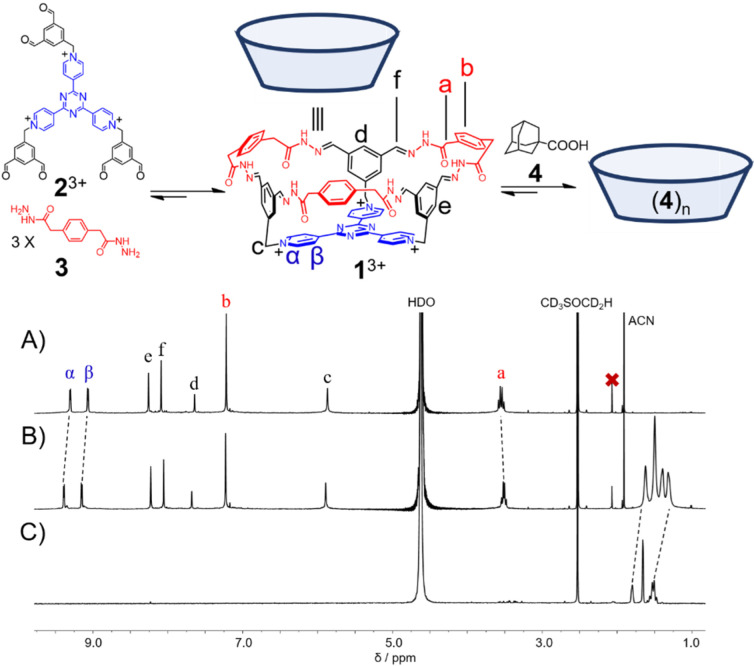
Partial ^1^H NMR spectra (500 MHz, 298 K, D_2_O/DMSO-d_6_ = 9:1, pD = 3) of **1**^3+^·3Cl^−^ (**A**) before and (**B**) after addition of guest **4**, and (**C**) guest **4**. In both (**B**,**C**), excess amount of **4** was suspended in solutions, guaranteeing that it is saturated.

A tricationic hexaformyl compound **2**^3+^·3Cl^−^ (1.0 mM) and a bishydrazide **3** (3.0 mM) was combined in D_2_O at room temperature. After 4 h, the ^1^H NMR spectrum (Figure 1A) was recorded, in which a set of simple resonances was observed, indicating that a highly symmetrical product, namely a bowl-shaped molecule **1**^3+^·3Cl^−^ was obtained in a [1 + 3] condensation manner. The resonance of the methylene unit *a* splits into two peaks, indicating that within the framework of **1**^3+^, the two protons in each of the methylene units become diastereotopic. The successful self-assembly of the bowl **1**^3+^ was further convinced by high resolution electrospray ionization mass spectrometry (HR-ESIMS). Two peaks were observed at m/z = 437.1750 and 673.2422, corresponding to the molecular cations of the bowl without and with one counteranion, namely [1]^3+^ and [**1** + Cl]^2+^, respectively (Figure S7, Supplementary Materials). **1**^3+^·3Cl^−^ was isolated in a 30% yield as a pure solid sample by means of counteranion exchange. However, the isolated pure **1**^3+^·3Cl^−^ is only sparingly soluble in water, whose solubility may be improved by addition of DMSO.

**Figure 2:**
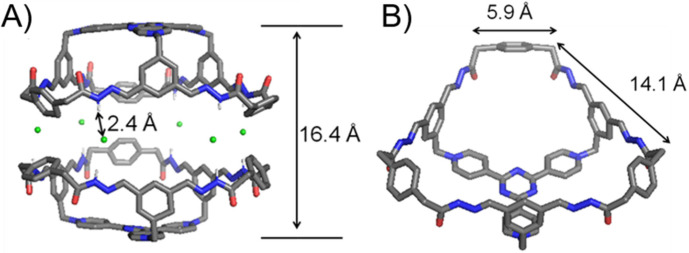
The (**A**) side on view and (**B**) top view of the single-crystal X-ray diffraction structures of **1**^3+^·3Cl^−^. C = grey, H = white, O = red, N = blue, Cl = green. Disordered solvent molecules are omitted for the sake of clarity. Only amide protons are shown because they are engaged in strong hydrogen bonding interactions.

Single crystals of **1**^3+^·3Cl^−^ were obtained by slowly diffusing dioxane into an aqueous solution of the self-assembled product. Single-crystal X-ray diffraction analysis unambiguously convinces the formation of the bowl-shaped host **1**^3+^ with a C_3v_ symmetry (Figure 2). The plane defined by each of the three phenyl “walls” in the **2**^3+^ residue orientates in an almost perpendicular manner, with respect to the tri(4-pyridyl)triazinyl (**TPT**) “platform”. The three phenyl “walls” are bridged with each other by three **3** residues. The upper rim of the bowl thus forms a large hexagonal opening, in which the longer and shorter edges are 14.1 and 5.9 Å, respectively (Figure 2B). The bowl **1**^3+^ features three approximately pentagonal windows. Both the imine and amide protons point to a direction away from the bowl cavity, allowing them to form hydrogen bonds with the Cl^−^ counteranions. Six Cl^−^ counteranions insert into the space between the amide rims of the two bowl molecules. The occurrence of hydrogen bonds is convinced by the corresponding close contacts, i.e., Cl−H_amide_ distances are around 2.4 Å. Driven by hydrogen bonds, two bowl molecules thus form a triangular prismatic bowl dimer with a D_3h_ symmetry. The two **TPT** platforms are separated by a distance of 16.4 Å. The volume of cavity of a bowl dimer is estimated to around 2000 Å^3^ (regard the cavity as a hexagonal prism approximately).

The capability of **1**^3+^ to accommodate guests in water was then investigated. Upon addition of a guest **4**, namely 1-adamantanecarboxylic acid, the resonances of the bowl **1**^3+^ recorded in aqueous DMSO (D_2_O/DMSO-d_6_ = 9:1) underwent modest shifts (Figure 1B). NOESY cross peaks (Figure S12, Supplementary Materials) between proton signals of **1**^3+^ and **4** unambiguously shows their complexation in solution. Only one set of resonances were observed corresponding to both the bowl and the guest, indicating that the host-guest complex undergoes relatively fast exchange with the their “free” states on the ^1^H NMR timescale. Since guest **4** (pKa = 4.9) is sensitive to pH changes, we also conducted the titration experiments in acidic (pD = 3) and basic conditions (pD = 9). In pD = 3 aqueous DMSO solution, the changes in the chemical shifts of the bowl **1**^3+^ were similar to that of the changes recorded in non-buffered aqueous DMSO solution. For example, the resonances of the pyridinium protons in the **TPT** base of **1**^3+^ were observed to undergo downfield shifts by around 0.1 ppm. In pD = 9 aqueous DMSO solution, however, the chemical shifts of the host remained unchanged upon the titration of **4**, and **1**^3+^ started to precipitate out when more than 1.5 equiv. of **4** was introduced, as the signals of **1**^3+^ gradually decreased during titration. The resonances of the guests underwent upfield shifts in both cases, indicating that the guests were encapsulated within the bowl cavity which provided a shielded magnetic environment (Figure 1B). We envisioned that in basic conditions where **4** exists in its deprotonated form, binding of the first guest is thermodynamically favored due to hydrophobic interaction as well as Coulombic attraction between host and guest. Nevertheless, binding of the second guest is inhibited due to repulsion between negatively charged guest molecules. The mismatch between the host cavity (~1000 Å^3^) and the deprotonated guest volume (172 Å^3^) [74] results in a very weak binding, which explains the insignificant change in the resonance of the bowl **1**^3+^ in basic conditions. In acidic or unbuffered aqueous solutions where the majority of **4** exists in its neutral form, binding of more than one guest molecule is possible. Due to the insufficient solubility of both host and guest as well as their weak association constant, the attempts to accurately determine the guest/host binding stoichiometry by using Job plot were unsuccessful [75]. However, based on the C_3v_ symmetry of the host cavity and Rebek’s 55% rule [76], we assumed that the bowl-shaped cavity of **1**^3+^ can accommodate up to three molecules of guest **4**, and such “fully” filled complexation can lead to the observable shifts of the proton signals of the molecular bowl upon addition of **4**.

## 3. Materials and Methods

All reagents and solvents were purchased from commercial sources and used without further purification. Manipulations were performed under a normal laboratory atmosphere unless otherwise noted. Nuclear magnetic resonance (NMR) spectra were recorded at ambient temperature using Bruker AVANCE III 400/500 or Agilent DD2 600 spectrometers, with working frequencies of 400/500/600 and 100/125/150 MHz for ^1^H and ^13^C, respectively. Chemical shifts are reported in ppm relative to the residual internal non-deuterated solvent signals (CDCl_3_: *δ*_H_ *=* 7.26 ppm, *δ*_C_ *=* 77.16 ppm, D_2_O: *δ*_H_ *=* 4.79 ppm, DMSO-d_6_: *δ*_H_ *=* 2.50 ppm, *δ*_C_ *=* 39.52 ppm). High-resolution mass spectra (HRMS) were measured using a SHIMADZU liquid chromatograph mass spectrometry ion trap time of flight (LCMS-IT-TOF) instrument. X-Ray crystallographic data were collected on a Bruker APEX-II CCD diffractometer.

## 4. Conclusions

In summary, a bowl-shaped molecule was successfully self-assembled by condensing a tricationic hexaaldehyde and three bishydrazide linker in water. The molecular bowl is composed of a planar triscationic base, on which a triangular rim containing six acylhydrazone functions is grafted. Taking advantage of hydrophobic effect, the cavity of this bowl is able to accommodate a hydrophobic guest in water. Using this bowl-shaped host as a molecular vessel to encapsulate substrates and catalyze their reactions are ongoing in our lab.

## Data Availability

Not applicable.

## Supplementary Materials

The following supporting information can be downloaded at: https://www.mdpi.com/article/10.3390/molecules28030976/s1. Scheme S1: Synthetic route of **2**^3+^·3Cl^−^; Figure S1: ^1^H NMR spectrum of **2**^3+^·3Cl^−^; Figure S2–S7: NMR characterizations of **1**^3+^·3Cl^−^; Figure S8: ESI-HRMS of **1**^3+^·3Cl−.; Figure S9: ^1^H NMR spectra of self-assembled products at different precursors concentrations; Figure S10–S11: ^1^H NMR titration of **1**^3+^·3Cl^−^ and **4** in non-buffered solution; Figure S12: NOESY spectrum of the host-guest complex; Figure S13–S16: ^1^H NMR titration of **1**^3+^·3Cl^−^ and **4** in buffer solutions; Figure S17: Different crystallographic views of **1**^3+^·3Cl^−^ [77,78,79].
