# Supplementary material for: Self-Assembly of a Purely Organic Bowl in Water via Acylhydrazone Formation
Source: Molecules. 2023 Jan 18;28(3):976. doi: 10.3390/molecules28030976 (PMC9921396; doi:10.3390/molecules28030976)
Supplement: Supplementary file 1 [file molecules-28-00976-s001.zip › molecules-2115280-supplementary.pdf]

Self-assembly of a purely organic bowl in water via acylhydrazone formation

Guangcheng Wu,[a,‡] Tianyu Jiao,[a,‡] and Hao Li[a,*]

[a]Department of Chemistry, Zhejiang University, Hangzhou 310027, China
[‡]These authors contributed equally to this work.
*e-mail: lihao2015@zju.edu.cn.

Supporting Information

Table of Contents

1. Materials and general methods S3

2. Synthetic procedures S4

3. Characterizations of molecular bowl $1^{3+}\cdot3Cl^{-}$ S8

4. Yield calculation of $1^{3+}\cdot3Cl^{-}$ at different precursor concentrations S15

5. ^{1}H NMR titration experiments demonstrating the formation of host-guest complex between $1^{3+}\cdot3Cl^{-}$ and 1-adamantanecarboxylic acid S17

6. X-ray Crystallography S21

References S22

1. Materials and general methods

All reagents and solvents were purchased from commercial sources and used without further purification. The bishydrazide **3** was prepared according to literature procedures.[S1] Manipulations were performed under a normal laboratory atmosphere unless otherwise noted. Nuclear magnetic resonance (NMR) spectra were recorded at ambient temperature using Bruker AVANCE III 400/500 or Agilent DD2 600 spectrometers, with working frequencies of 400/500/600 and 100/125/150 MHz for ^{1}H and ^{13}C, respectively. Chemical shifts are reported in ppm relative to the residual internal non-deuterated solvent signals ($CDCl_3$: δ_H = 7.26 ppm, δ_C = 77.16 ppm, D_2O: δ_H = 4.79 ppm, DMSO-d_6: δ_H = 2.50 ppm, δ_C = 39.52 ppm). High-resolution mass spectra (HRMS) were measured using a SHIMADZU liquid chromatograph mass spectrometry ion trap time of flight (LCMS-IT-TOF) instrument. X-Ray crystallographic data were collected on a Bruker APEX-II CCD diffractometer.

2. Synthetic procedures

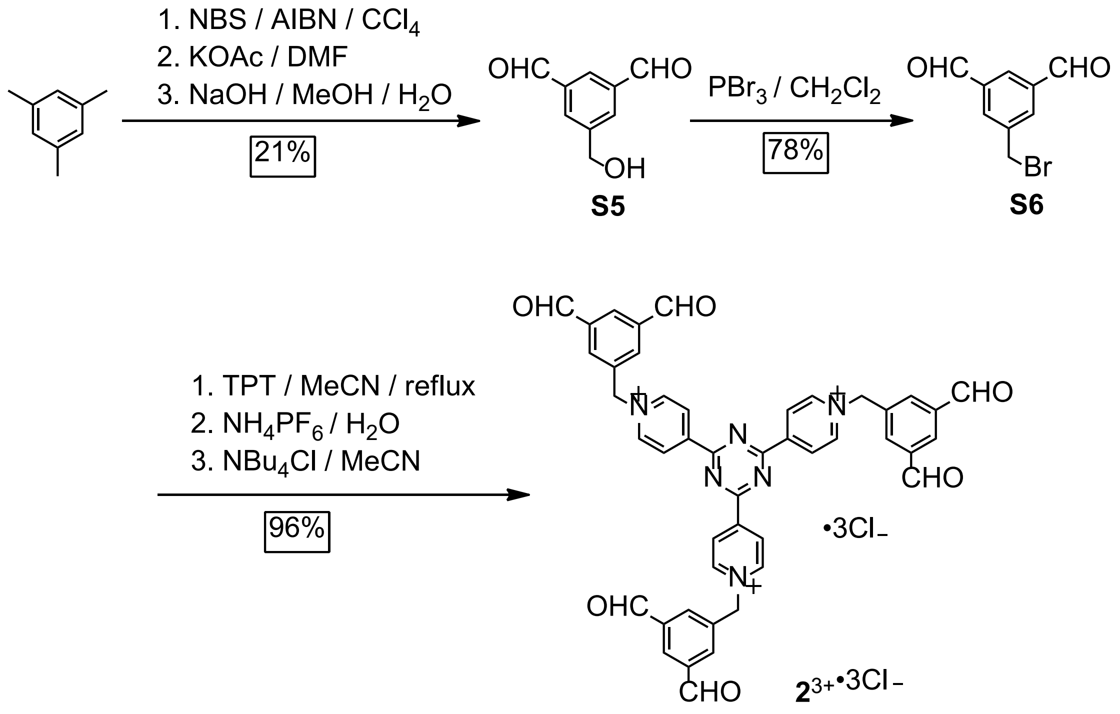

Scheme S1: Synthesis of **2**$^{3+}$·3Cl^{-}.

Compound S5: A mixture of mesitylene (10 g, 83 mmol), NBS (81.5 g, 0.46 mol), and AIBN (1.4 g, 8.3mmol) in CCl_4 (500 mL) was stirred and heated to reflux for 12 h. When the reaction was complete, removal of the solvent under reduced pressure afforded a yellow oil, to which KOAc (82 g, 0.83 mol) and DMF (200 mL) was then added. The reaction mixture was then stirred at 90ºC overnight. After removal of the solvent under reduced pressure, MeOH (30 mL), H_2O (30 mL), NaOH (4.8 g, 0.12 mol) were then added into the reaction residue, which was stirred at room temperature for 3 h. The reaction mixture was extracted with ethyl acetate and washed with brine, and the organic layer was dried over sodium sulfate. The solvent was then removed, affording the crude product, which was then purified by column chromatography on silica gel (Petroleum Ether /Ethyl Acetate = 3:1) to give **S5** as a white solid (2.9 g, 21%). ^{1}H NMR (500 MHz, 298K, $CDCl_3$) δ

= 10.12 (s, 2H), 8.29 (s, 1H), 8.17 (s, 2H), 4.90 (s, 2H); ^{13}C NMR (125 MHz, 298K, $CDCl_3$) δ = 191.2, 143.5, 137.4, 132.7, 130.2, 63.9. The ^{1}H NMR spectrum is consistent with the reported one.[S2]

Compound S6: S5 (1.64 g, 10.0 mmol) was dissolved in 20 mL DCM, to which PBr_3 (2.71 g, 10.0 mmol) was added. The reaction mixture was stirred at r.t. for 2 h. The reaction mixture was extracted with DCM and washed with water. The organic layer was dried over sodium sulfate. Removal of the solvent afforded the crude product, which was then purified by column chromatography on silica gel (Petroleum Ether /Ethyl Acetate = 3:1) to give **S6** as a white solid (1.77 g, 78%). ^{1}H NMR (500 MHz, 298K, $CDCl_3$) δ = 10.10 (s, 2H), 8.30 (s, 1H), 8.16 (s, 2H), 4.58 (s, 2H).

Compound $2^{3+}\cdot 3Cl^{-}$: A mixture of **S6** (1.77 g, 7.80 mmol) and tri(4-pyridyl)triazine (0.162 g, 0.520 mmol) was dissolved in MeCN (15 mL) and heated to reflux for 10 days, during which yellow precipitates were observed. After cooling to room temperature, the precipitates were collected by filtration to afford the crude product as yellow solids. The solid was then dissolved in water, to which a saturated NH_4PF_6 solution was added until precipitation was complete. The white precipitate was collected via filtration and dissolved in MeCN. NBu_4Cl was then added, yielding pale yellow precipitates. The precipitates were then collected by filtration and washed with MeCN, yielding **2**$^{3+}\cdot 3Cl^{-}$ (0.429 g, 96%) without further purification. ^{1}H NMR (400 MHz, 298K, DMSO-*d*$_6$) δ = 10.15 (s, 6H), 9.76 (d, *J* = 6.8 Hz, 6H), 9.58 (d, *J* = 6.8 Hz, 6H), 8.55 (s, 3H), 8.51 (s, 6H), 6.29 (s, 6H); ^{13}C NMR (100 MHz, 298K, DMSO-*d*$_6$) δ = 192.1, 168.7, 148.6, 146.8, 137.4, 136.4, 134.5, 132.2, 127.5, 62.4; HRMS (ESI, H_2O) m/z [**2**]$^{3+}$ calcd for $C_{45}H_{33}N_6O_6^{3+}$: 251.0815; found 251.0826.

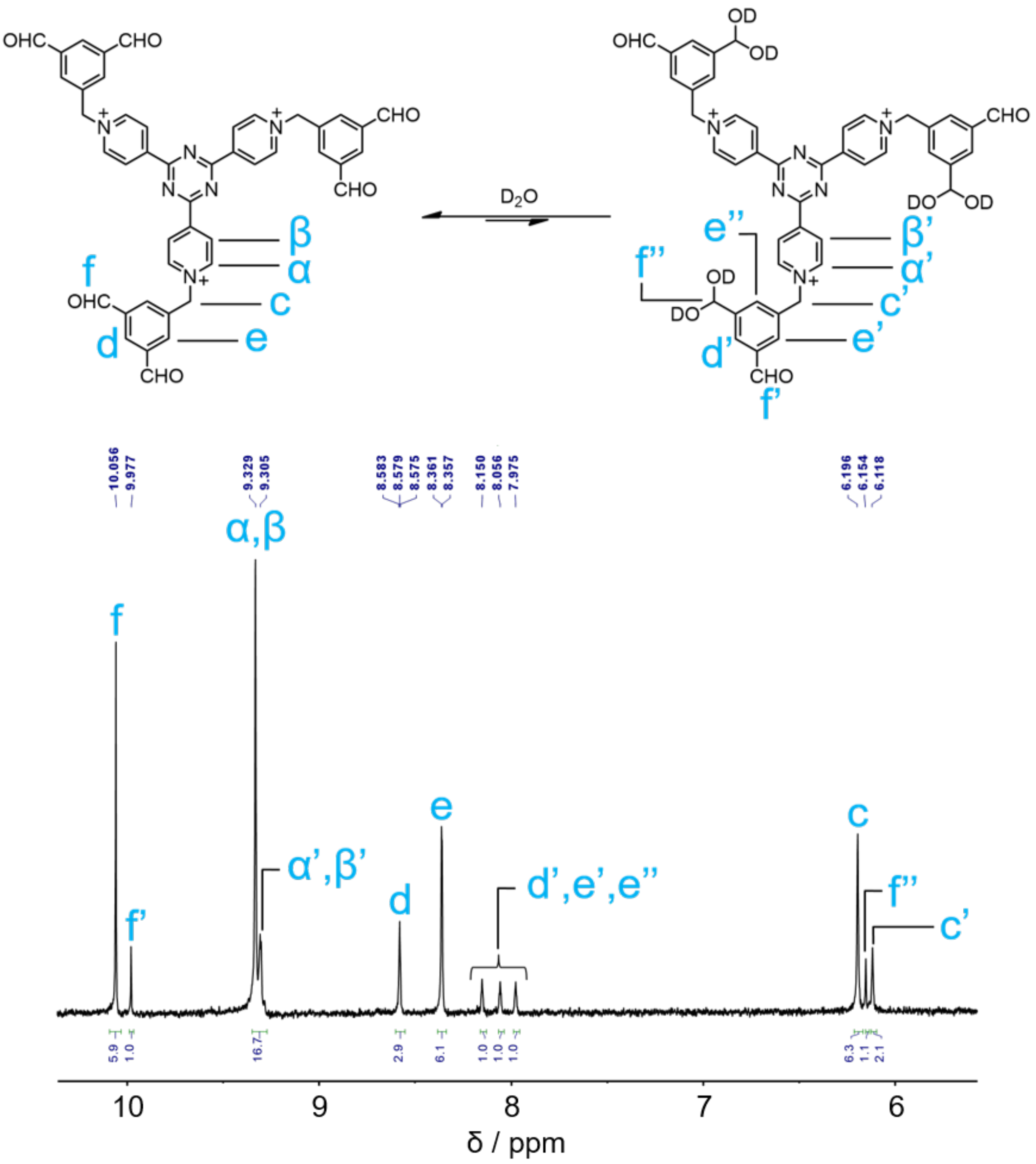

Figure S1: ^{1}H NMR (400 MHz, 298K, D_2O) of $\mathbf{2}^{3+}\cdot 3Cl^-$. In D_2O solution, around 25% of the formyl functions in $\mathbf{2}^{3+}\cdot 3Cl^-$ underwent partial hydration.

Compound $\mathbf{1}^{3+}\cdot 3Cl^-$: A mixture of $\mathbf{2}^{3+}\cdot 3Cl^-$ (43.0 mg, 0.050 mmol) and **3** (35.5 mg, 0.160 mmol) was dissolved in water (100 mL), and catalytic amount of TFA (5 μmol) was added. The reaction mixture was heated to 50 °C for 5 hours, followed by the addition of 2 g of NH_4PF_6. The resulting precipitate was separated by centrifugation, washed with water and dried in vacuum, which was then suspended in MeCN. To this MeCN suspension was added NBu_4Cl, leading to the precipitation

of $\mathbf{1}^{3+}\cdot 3Cl^{-}$ (21.3 mg, 30%), which was separated by filtration and washed with MeCN. Product obtained by this method is only sparingly soluble in water, and DMSO may improve its dissolution. Another way for purification is to use reverse phase HPLC (C18 column, H_2O/ACN, 0.1% TFA added) to separate the product from the self-assembled mixture, which provides product with better water solubility. Characterizations of $\mathbf{1}^{3+}\cdot 3Cl^{-}$ will be provided in the next section.

3. Characterizations of molecular bowl $1^{3+}\cdot 3Cl^{-}$

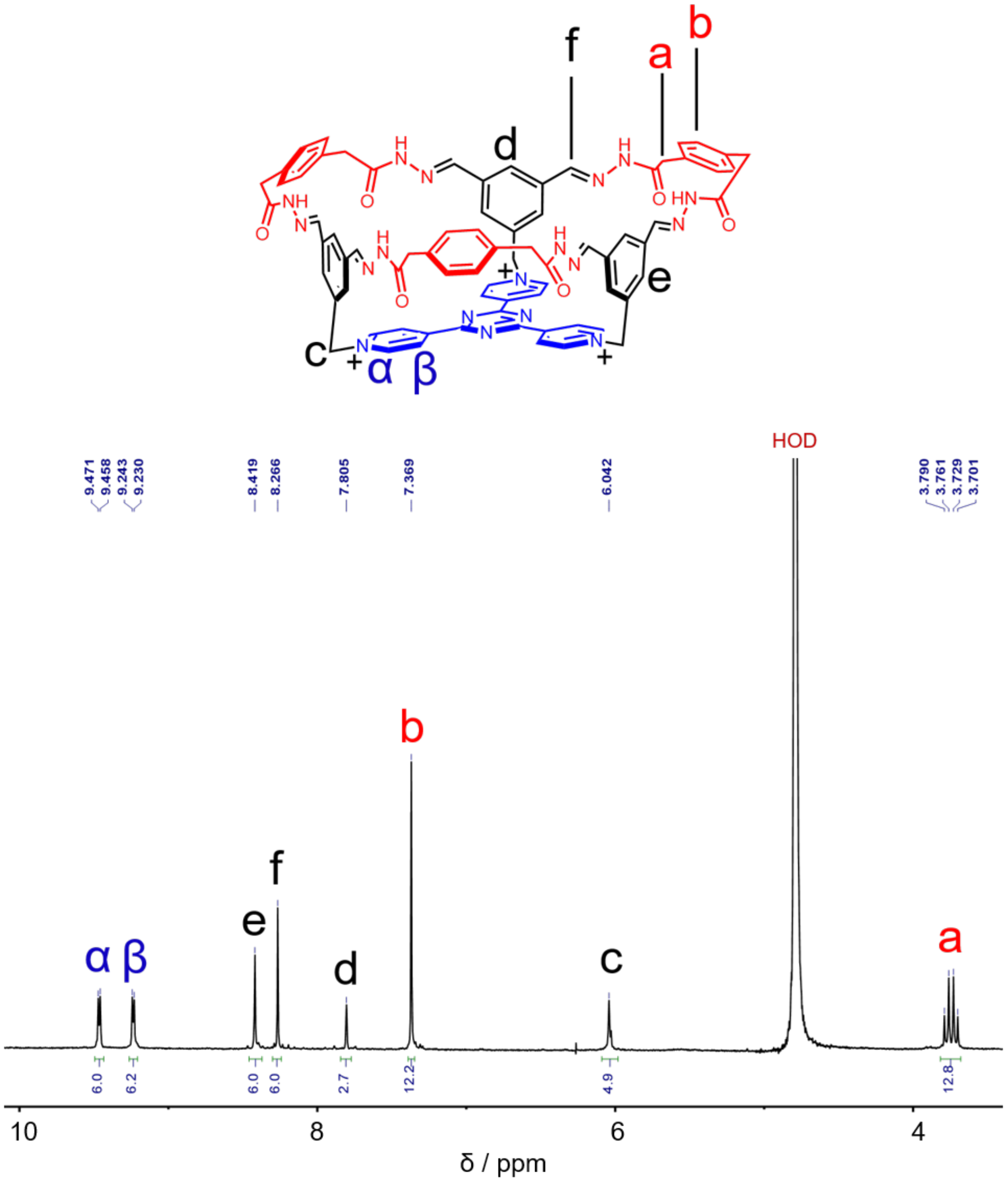

Figure S2: ^{1}H NMR (500 MHz, 298K, D_2O) of $\mathbf{1}^{3+}\cdot 3Cl^{-}$.

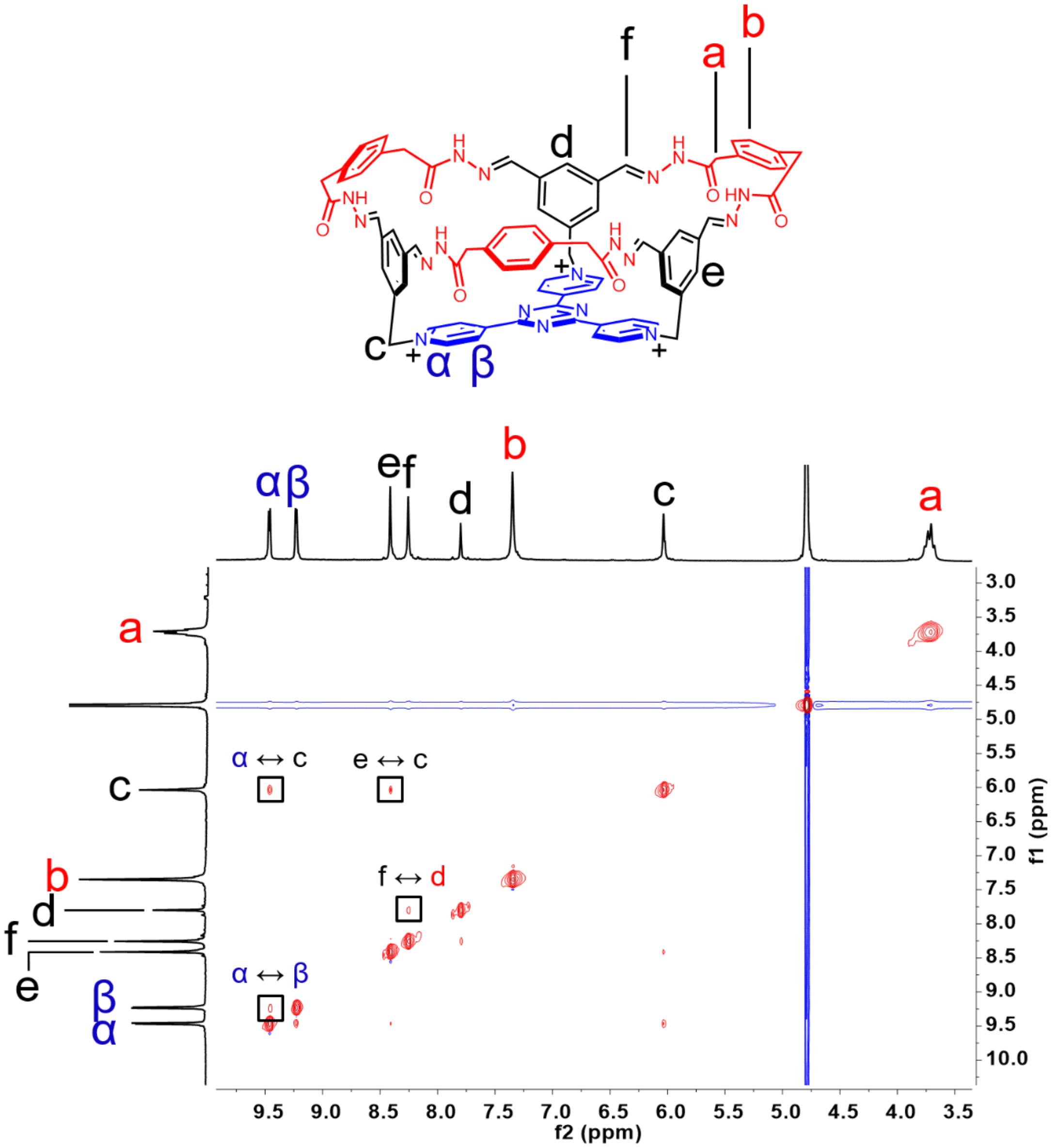

Figure S3: ^{1}H-^{1}H COSY spectrum (500 MHz, 298 K, D_2O) of $\mathbf{1}^{3+}\cdot 3Cl^{-}$. Key correlation peaks are labeled.

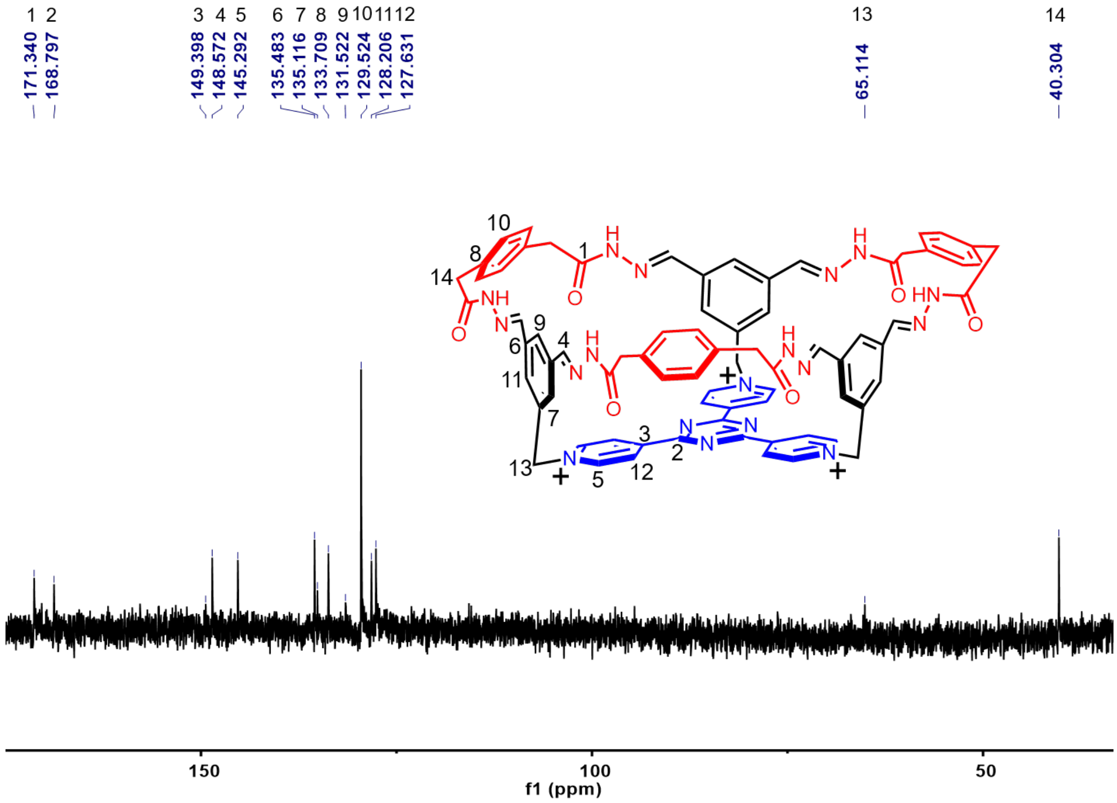

Figure S4. ^{13}C NMR spectrum (125 MHz, 298 K, D_2O) of $\mathbf{1}^{3+}\cdot 3Cl^{-}$.

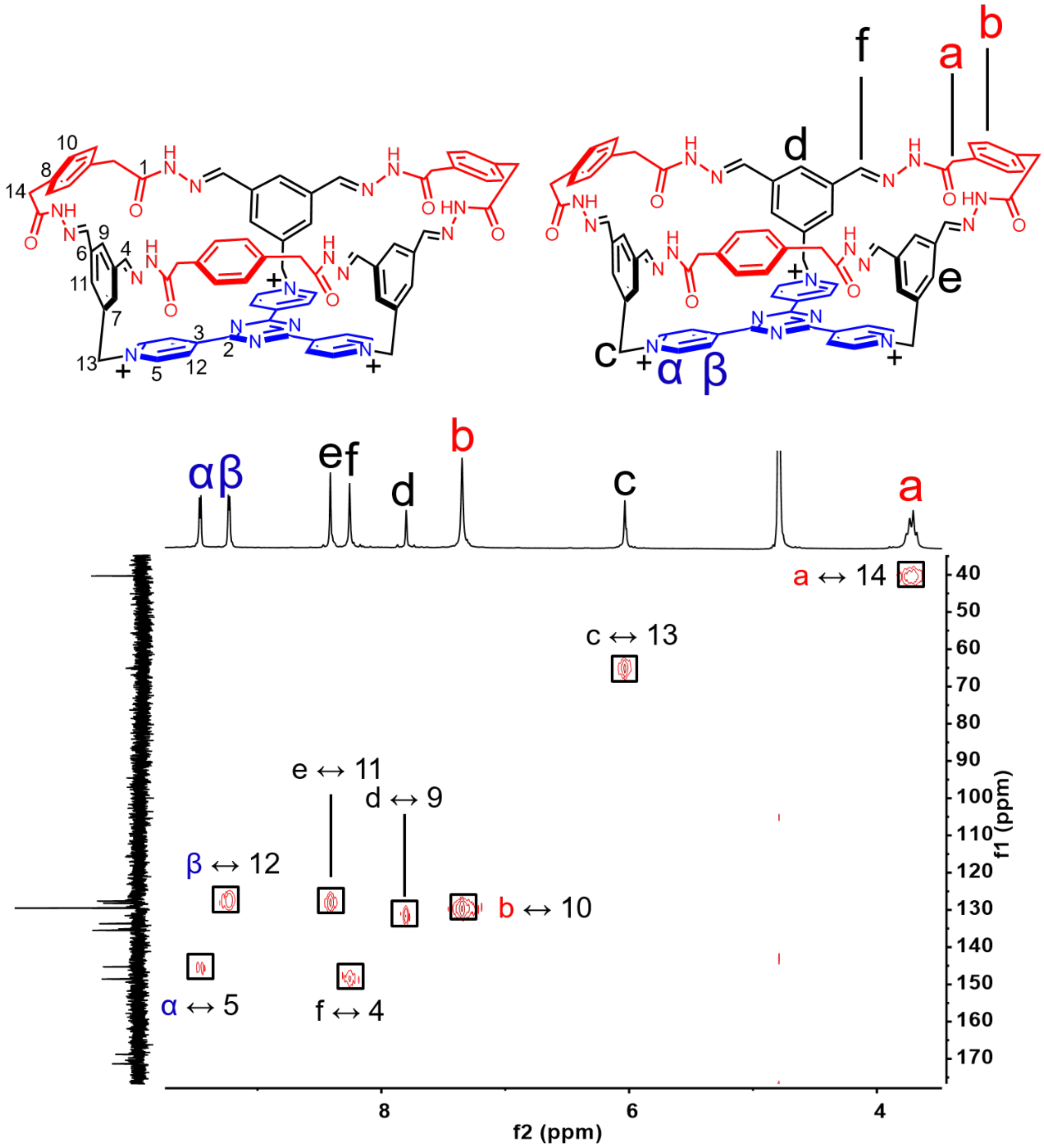

Figure S5: HSQC spectrum (298 K, D_2O) of $\mathbf{1}^{3+}\cdot 3Cl^-$. Key correlation peaks are labeled.

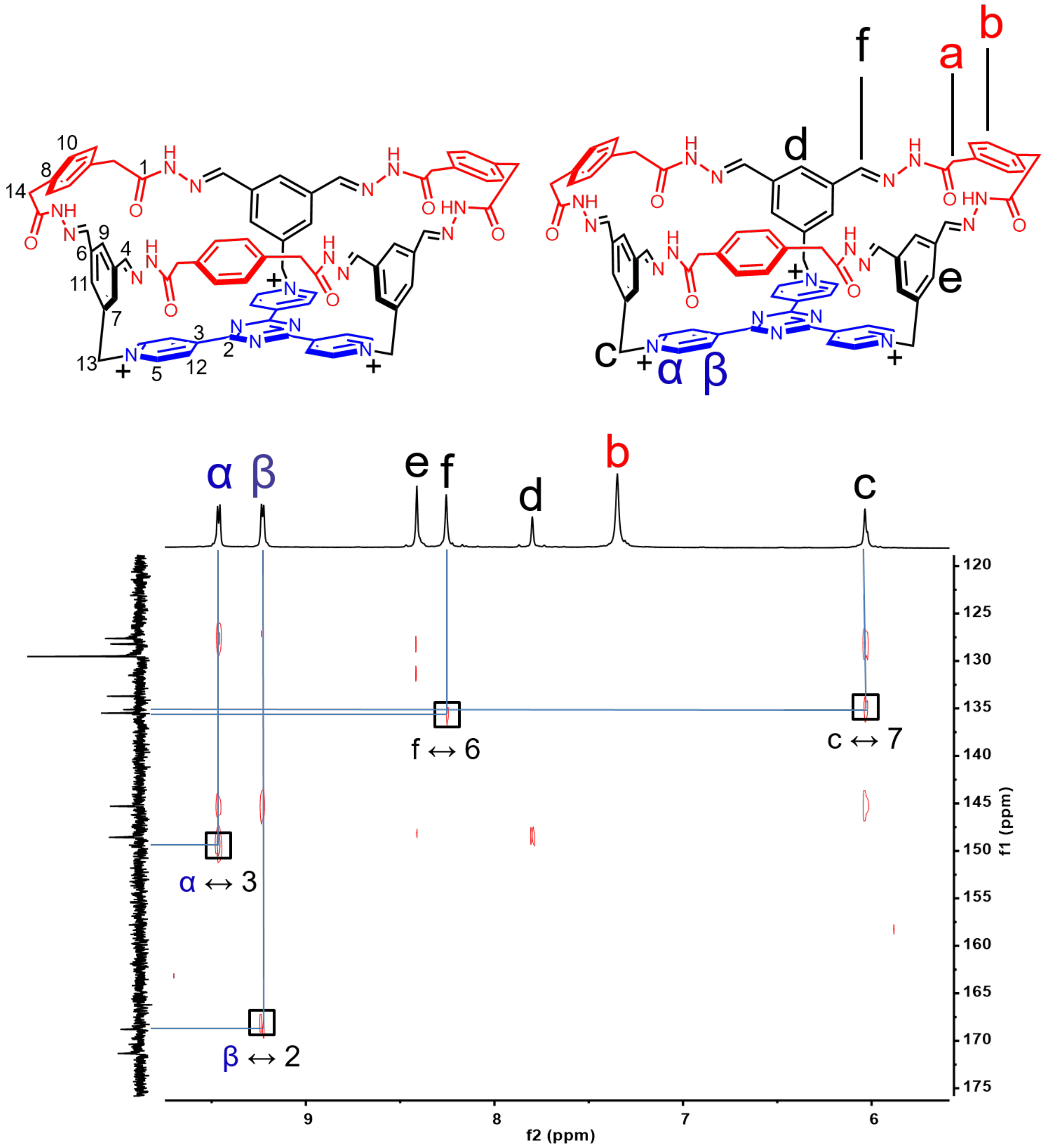

Figure S6: Partial HMBC spectrum (298 K, D_2O) of **1**$^{3+}$·3Cl^{-}. Key correlation peaks are labeled.

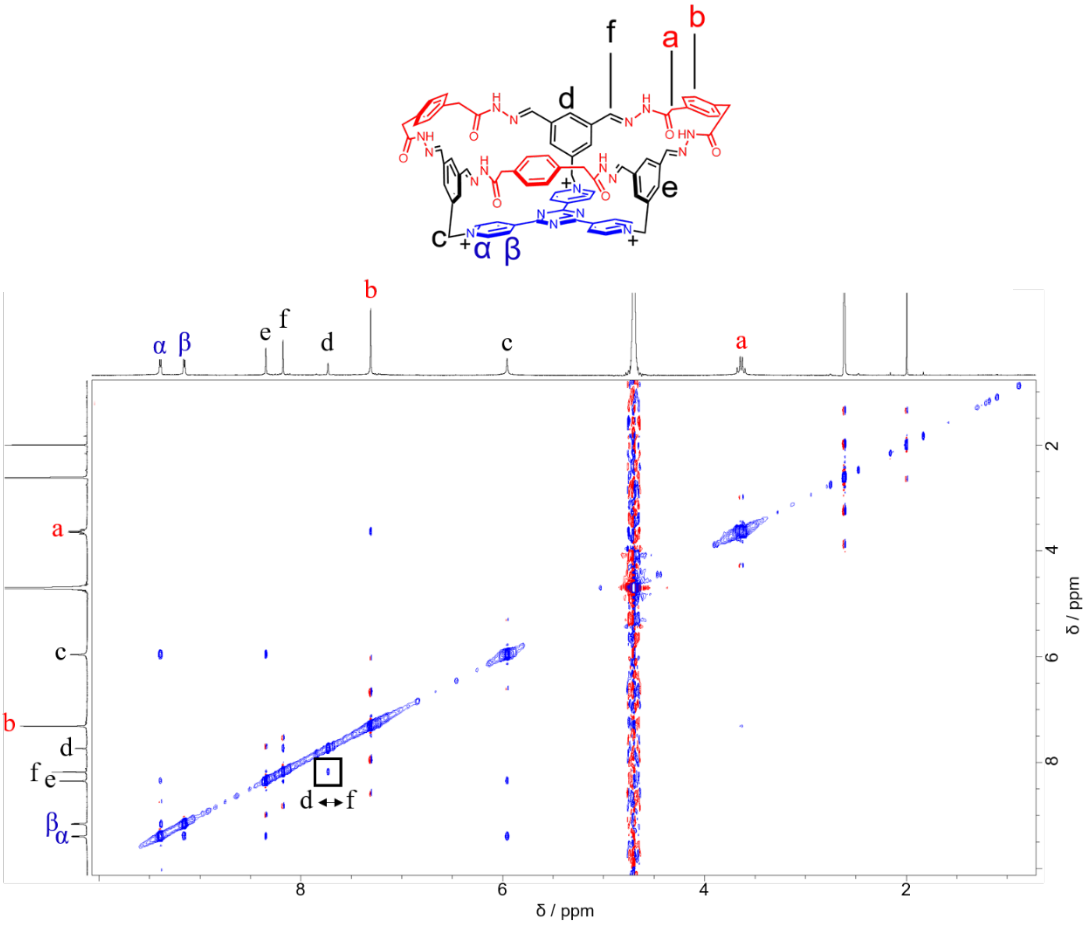

Figure S7: ^{1}H-^{1}H NOESY spectrum (500MHz, 298 K, D_2O/DMSO-d_6 = 9:1) of $\mathbf{1}^{3+}\cdot 3Cl^-$. Key correlation peaks are labeled.

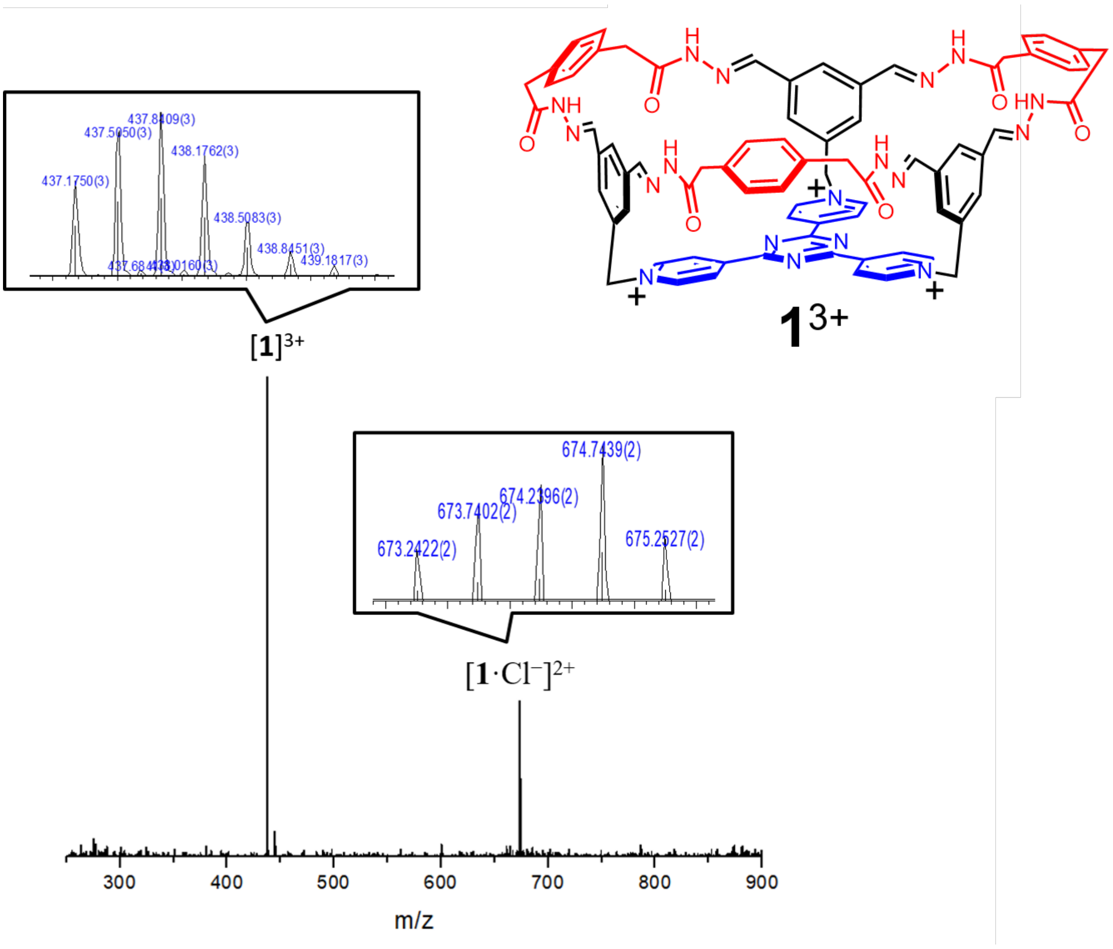

Figure S8. ESI-HRMS of $\mathbf{1}^{3+}\cdot 3Cl^{-}$. *m/z* $[\mathbf{1}]^{3+}$ calcd for $C_{75}H_{63}N_{18}O_6^{3+}$: 437.1721; found: 437.1750. $[\mathbf{1}\cdot Cl^{-}]^{2+}$ calcd for $C_{68}H_{63}N_{12}O_4Cl^{2+}$: 673.2428; found: 673.2422.

4. Yield calculation of $1^{3+}\cdot 3Cl^{-}$ at different precursor concentrations

In order to obtain the yields of $\mathbf{1}^{3+}\cdot 3Cl^{-}$ in the conditions of different concentrations of precursors, i.e., $\mathbf{2}^{3+}\cdot 3Cl^{-}$ and **3**, a D_2O solution comprising of 2.35mM of $\mathbf{2}^{3+}\cdot 3Cl^{-}$, 7.05 mM of **3** and 11.0 mM of EtOH was carefully prepared, and through dilution with D_2O, four other solutions containing 0.46, 0.70, 1.05 and 1.57 mM of $\mathbf{2}^{3+}\cdot 3Cl^{-}$ respectively were also obtained, so that in all five solutions, the molar ratio between $\mathbf{2}^{3+}\cdot 3Cl^{-}$, **3** and EtOH remained constant, namely, 1 / 3 / 4.69. Both solutions were heated to 50 ºC overnight before ^{1}H-NMR measurements. The integration proportion between resonances corresponding to $\mathbf{1}^{3+}\cdot 3Cl^{-}$ and EtOH was then used to calculate the concentration and thus the NMR yield of $\mathbf{1}^{3+}\cdot 3Cl^{-}$.

By this means, the self-assemble yields of $\mathbf{1}^{3+}\cdot 3Cl^{-}$ were calculated to be 95%, 88%, 74%, 57% and 33%, respectively, in the concentration of 0.46, 0.70, 1.05, 1.57 and 2.35 mM in terms of $\mathbf{2}^{3+}\cdot 3Cl^{-}$, respectively.

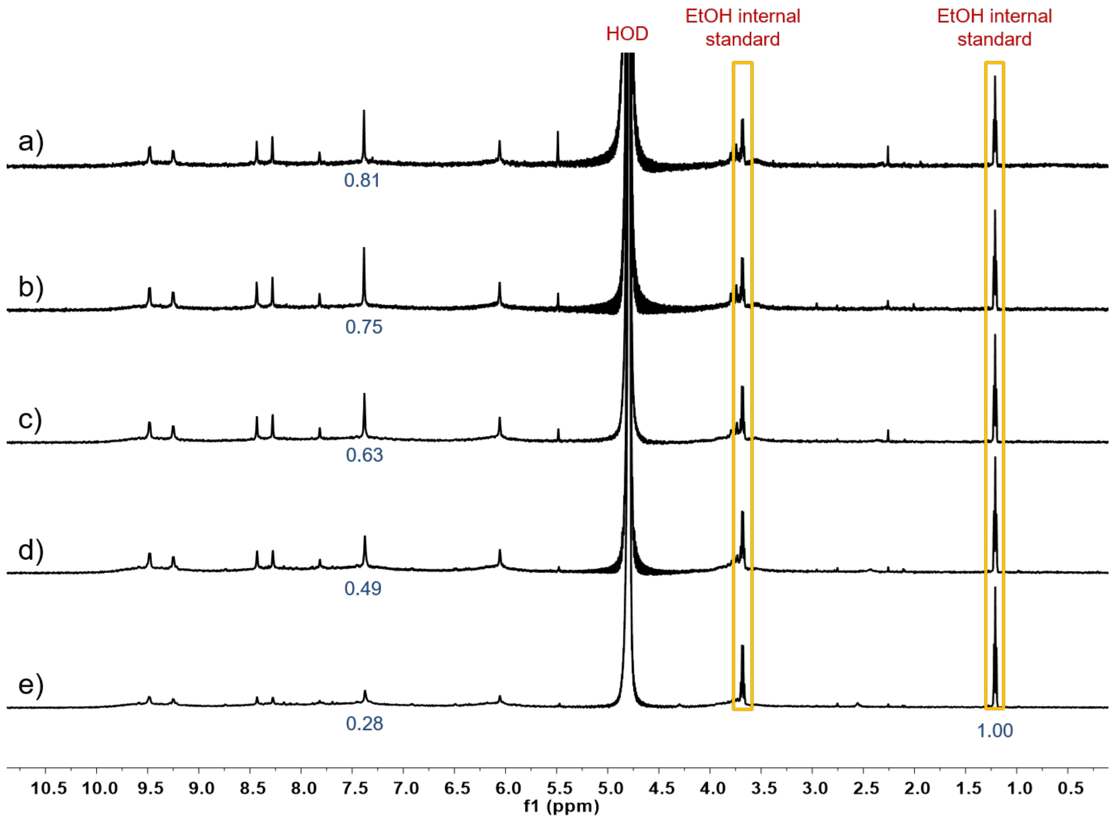

Figure S9: ^{1}H NMR spectra (600 MHz, 298 K, D_2O) of self-assembled products composing of different concentrations of $\mathbf{2}^{3+}\cdot 3Cl^{-}$ and **3**: (a) 0.46 mM, (b) 0.70 mM, (c) 1.05 mM, (d) 1.57 mM, (e) 2.35 mM in terms of $\mathbf{2}^{3+}\cdot 3Cl^{-}$.

5. ^{1}H NMR titration experiments demonstrating the formation of host-guest complex between $1^{3+}\cdot 3Cl^{-}$ and 1-adamantanecarboxylic acid

5.1) In non-buffered solution:

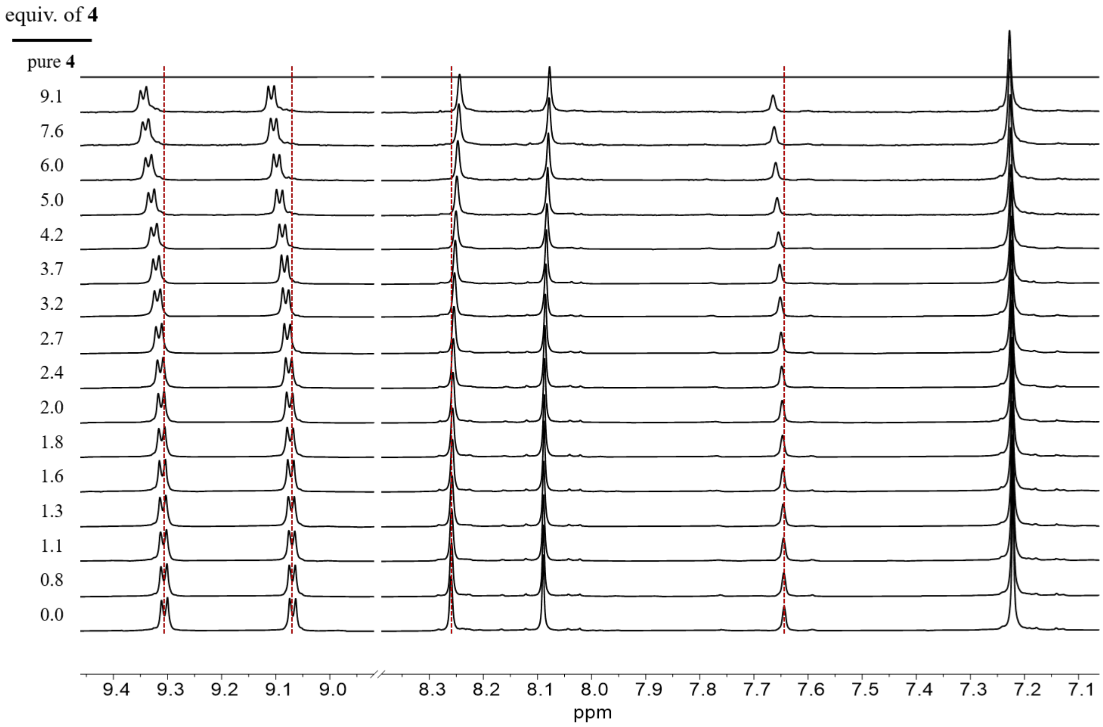

Figure S10: Partial ^{1}H NMR spectrum (600 MHz, D_2O/DMSO-d_6 = 9:1, 298 K) of $\mathbf{1}^{3+}\cdot 3Cl^{-}$ (0.113 mM) when titrated with **4**.

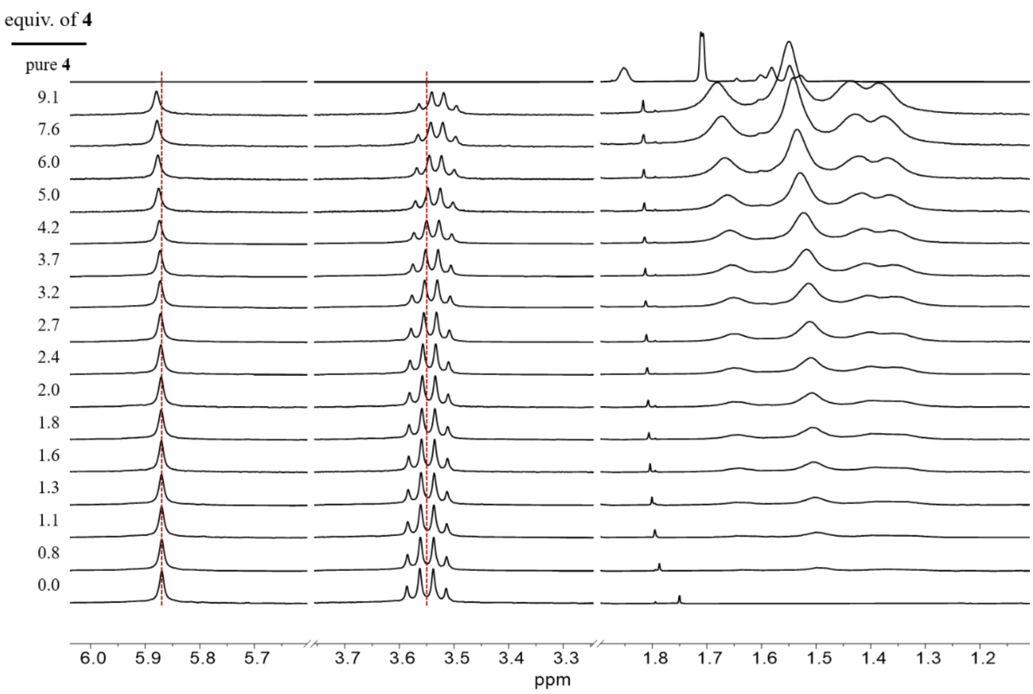

Figure S11: Partial ^{1}H NMR spectrum (600 MHz, D_2O/DMSO-d_6 = 9:1, 298 K) of $\mathbf{1}^{3+}\cdot 3Cl^{-}$ (0.113 mM) when titrated with **4**.

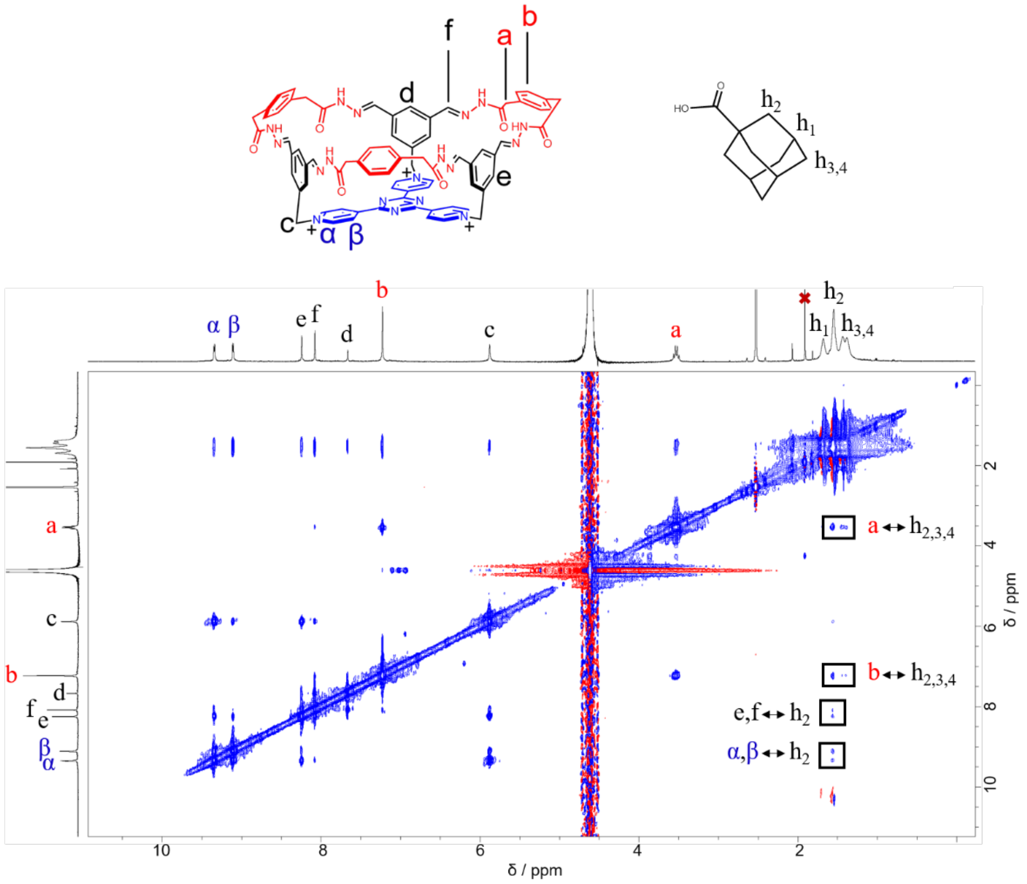

Figure S12: ^{1}H-^{1}H NOESY spectrum (600MHz, 298 K, D_2O/DMSO-d_6 = 9:1) of $\mathbf{1}^{3+}\cdot 3Cl^{-}$ with 9 equivalents of **4**. Key correlation peaks are labeled.

5.2) In acidic solution:

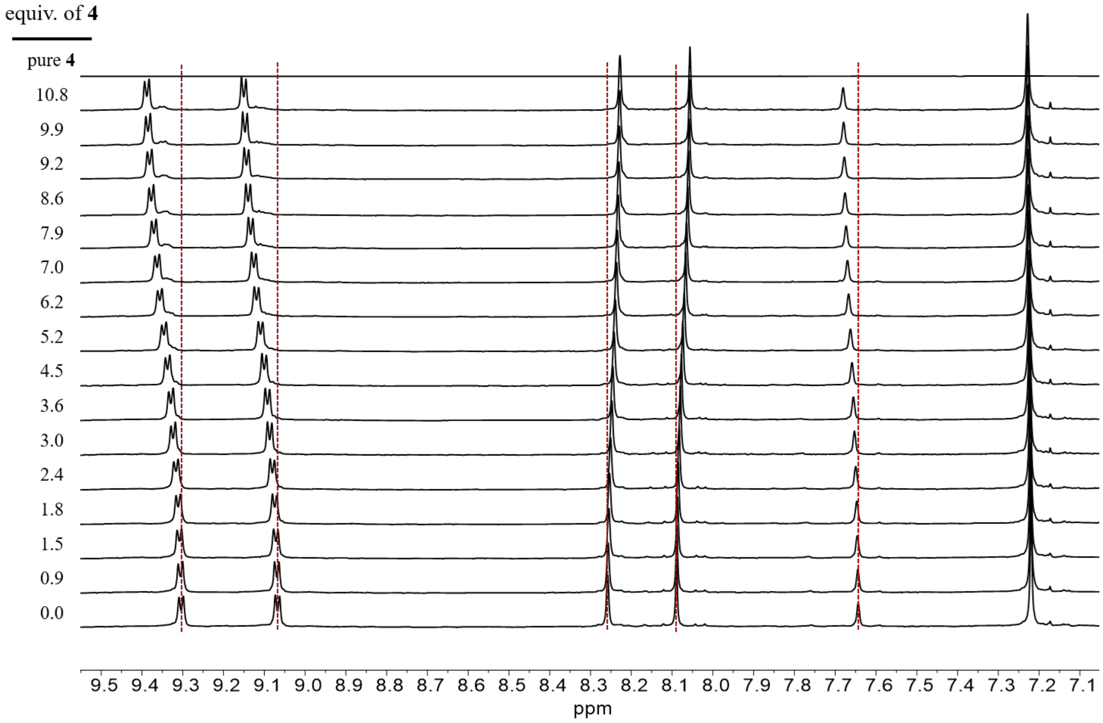

Figure S13: Partial ^1H NMR spectrum (600 MHz, D_2O/DMSO-d_6 = 9:1, 5 mM DCl, pD = 3, 298 K) of $\mathbf{1}^{3+}\cdot 3Cl^-$ (0.113 mM) when titrated with **4**.

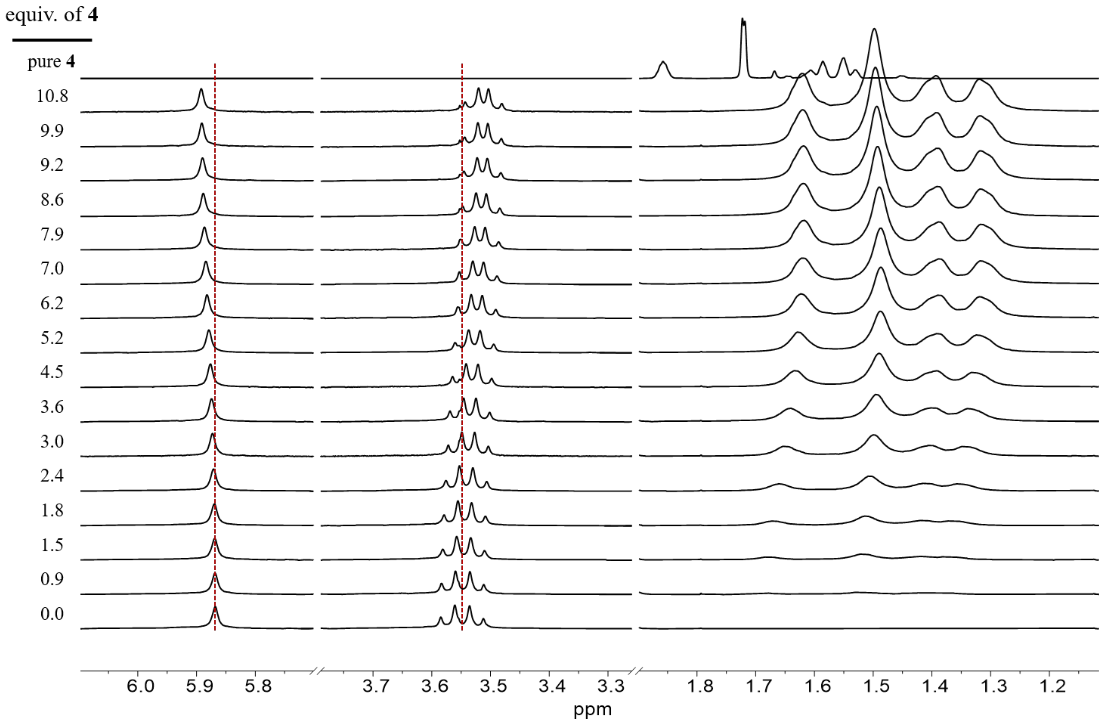

Figure S14: Partial ^1H NMR spectrum (600 MHz, D_2O/DMSO-d_6 = 9:1, 5 mM DCl, pD = 3, 298 K) of $\mathbf{1}^{3+}\cdot 3Cl^-$ (0.113 mM) when titrated with **4**.

5.3) In basic buffered solution:

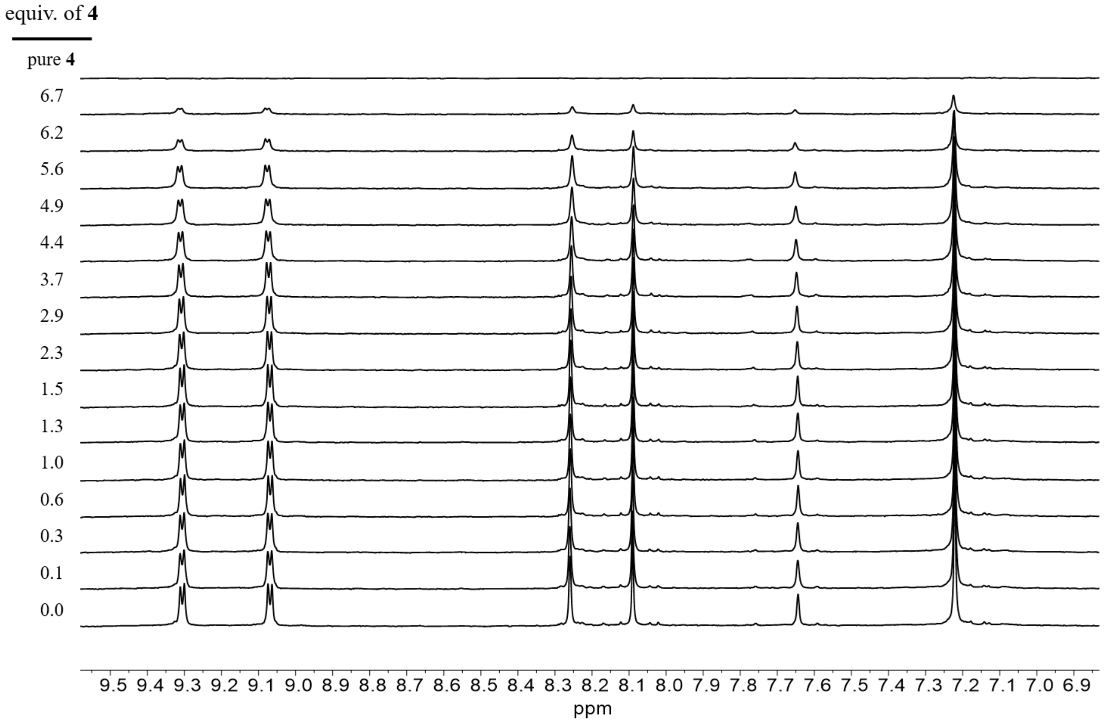

Figure S15: Partial ^{1}H NMR spectrum (600 MHz, D_2O/DMSO-d_6 = 9:1, 10 mM of NH_3-NH_4Cl buffer, pD = 9, 298 K) of $\mathbf{1}^{3+}\cdot 3Cl^{-}$ (0.113 mM) when titrated with **4**.

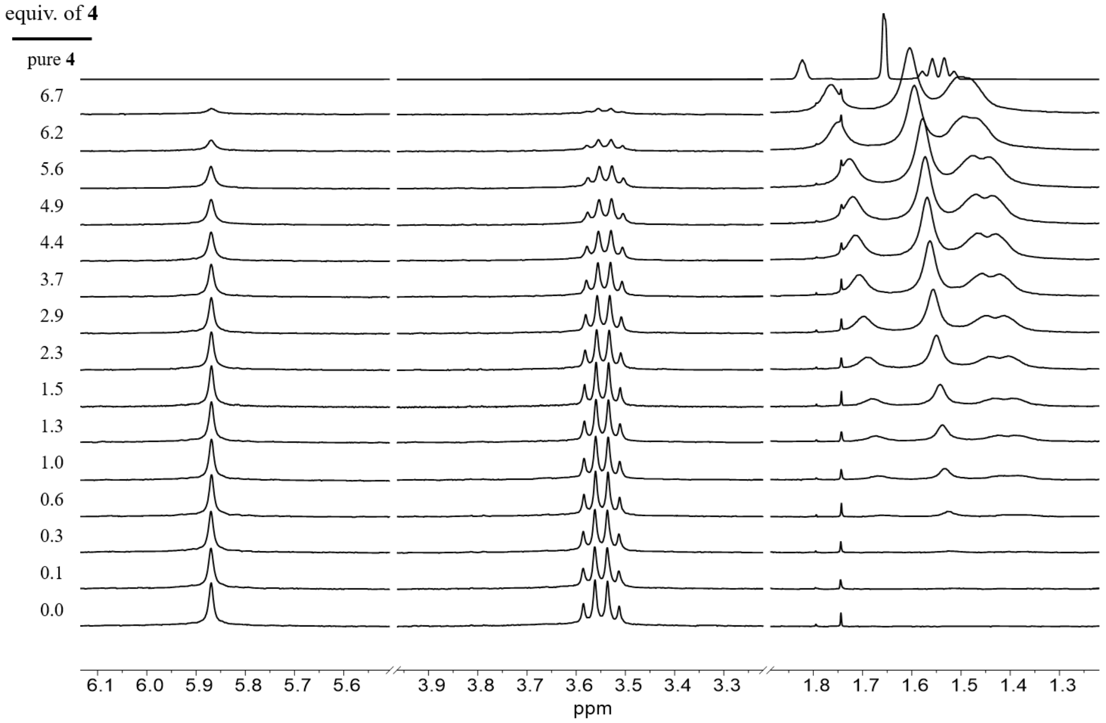

Figure S16: Partial ^{1}H NMR spectrum (600 MHz, D_2O/DMSO-d_6 = 9:1, 10 mM of NH_3-NH_4Cl buffer, pD = 9, 298 K) of $\mathbf{1}^{3+}\cdot 3Cl^{-}$ (0.113 mM) when titrated with **4**.

Addition of more than 1.5 eq. of **4** in basic conditions leads to precipitation of the bowl $\mathbf{1}^{3+}$.

6. X-ray Crystallography

6.1) Methods

Slow vapor diffusion of 1,4-dioxane into the solution of self-assembled $\mathbf{1}^{3+}\cdot 3Cl^{-}$ (around 1.0 mg/mL) in D_2O at room temperature over the period of 3 months yielded yellow single crystals of $\mathbf{1}^{3+}\cdot 3Cl^{-}$. X-Ray crystallographic data was collected on a Bruker APEX-II CCD diffractometer. The crystal was kept at 170 K during data collection. The structure was solved using Olex2 software[S3] with the ShelXS structure solution program by Direct Method and refined with the XL[S4] refinement package through Least Squares minimization.

6.2) Crystal Data

$(C_{37.5}H_{31.5}N_9O_3)_2\bullet(Cl)_3$, M = 1418.78, triclinic, space group $P\ n\ m\ a$, a = 21.914(2), b = 27.594(3), c = 45.201(5), $\alpha = \beta = \gamma = 90$, V = 27332.8(5) Å^3, Z = 8, T = 170 K, μ(GaKα) = 0.586 mm^{-1}, $Dcalc$ = 0.690 g/mm^3, 54935 reflections measured (6.19° ≤ 2Θ ≤ 75.13°), 10979 unique (R_{int} = 0.0841, R_{sigma} = 0.0951) which were used in all calculations. The final R_1 was 0.1637 (I>=2σ(I)) and wR_2 was 0.4673 (all data). CCDC number: 1946455.

6.3) Solid-State (-Super) structures

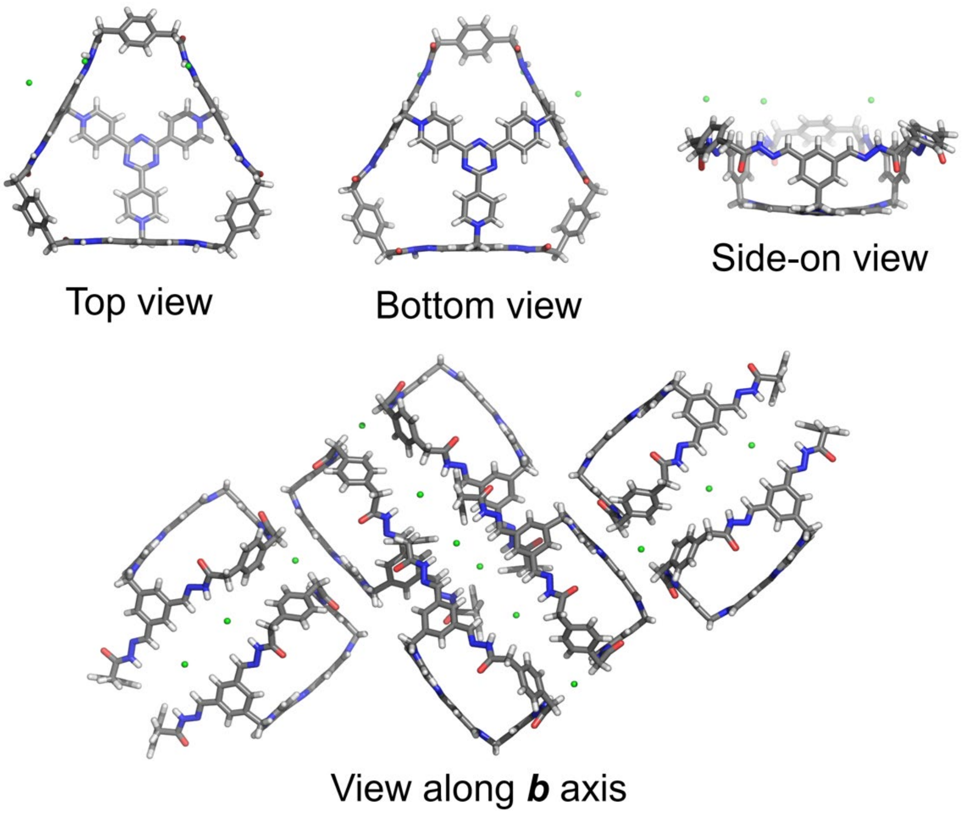

Figure S17. Different crystallographic views of $\mathbf{1}^{3+}\cdot 3Cl^{-}$.

References

S1 C. Y. Wang, G. Wu, T. Jiao, L. Shen, G. Ma, Y. Pan and H. Li, *Chem. Commun.*, 2018, **54**, 5106-5109.

S2 G. R. Newkome, T. J. Cho, C. N. Moorefield, R. Cush, P. S. Russo, L. A. GodÌnez, M. J. Saunders and P. Mohapatra, *Chem. Eur. J.*, 2002, **8**, 2946-2954.

S3 O. V. Dolomanov, L. J. Bourhis, R. J. Gildea, J. A. K. Howard and H. Puschmann, *J. Appl. Crystallogr.*, 2009, **42**, 339-341.

S4 G. M. Sheldrick, *Acta. Crystallogr. A*, 2008, **64**, 112-122.
